# The *Bunocephalus coracoideus* Species Complex (Siluriformes, Aspredinidae). Signs of a Speciation Process through Chromosomal, Genetic and Ecological Diversity

**DOI:** 10.3389/fgene.2017.00120

**Published:** 2017-09-21

**Authors:** Milena Ferreira, Caroline Garcia, Daniele A. Matoso, Isac S. de Jesus, Marcelo de B. Cioffi, Luiz A. C. Bertollo, Jansen Zuanon, Eliana Feldberg

**Affiliations:** ^1^Laboratório de Genética Animal, Coordenação de Biodiversidade, Instituto Nacional de Pesquisas da Amazônia Manaus, Brazil; ^2^Laboratório de Citogenética, Departamento de Ciências Biológicas, Universidade Estadual do Sudoeste da Bahia Jequié, Brazil; ^3^Laboratório de Citogenômica Animal, Instituto de Ciências Biológicas, Departamento de Genética, Universidade Federal do Amazonas Manaus, Brazil; ^4^Laboratório de Fisiologia Comportamental e Evolução, Coordenação de Biodiversidade, Instituto Nacional de Pesquisas da Amazônia Manaus, Brazil; ^5^Departamento de Genética e Evolução, Universidade Federal de São Carlos São Carlos, Brazil; ^6^Laboratório de Sistemática e Ecologia de Peixes, Coordenação de Biodiversidade, Instituto Nacional de Pesquisas da Amazônia Manaus, Brazil

**Keywords:** chromosomal differentiation, molecular taxonomy, ecological adaptations, evolutionary units, banjo catfish

## Abstract

*Bunocephalus* is the most species-rich Aspredinidae genus, corresponding to a monophyletic clade with 13 valid species. However, many species have their classification put in question. Here, we analyzed individuals from four Amazonian populations of *Bunocephalus coracoideus* by cytogenetic and molecular procedures. The geographic distribution, genetic distances and karyotype data indicate that each population represents an Evolutionary Significant Unit (ESU). Cytogenetic markers showed distinct 2n and karyotype formulas, as well as different numbers and locations of the rDNA sites among ESUs. One of such populations (ESU-D) highlighted an extensive polymorphic condition, with several cytotypes probably due to chromosomal rearrangements and meiotic non-disjunctions. This resulted in several aneuploid karyotypes, which was also supported by the mapping of telomeric sequences. Phylograms based on Maximum Likelihood (ML) and Neighbor Joining (NJ) analyses grouped each ESU on particular highly supported clades, with the estimation of evolutionary divergence indicating values being higher than 3.8–12.3% among them. Our study reveals a huge degree of chromosomal and genetic diversity in *B. coracoideus* and highly points to the existence of four ESUs in allopatric and sympatric speciation processes. In fact, the high divergences found among the ESUs allowed us to delimitate lineages with taxonomic uncertainties in this nominal species.

## Introduction

*Bunocephalus* is the most species-rich Aspredinidae genus, corresponding to a monophyletic clade (Cardoso, 2008, 2010) with 13 valid species (Eschmeyer et al., 2017). However, many species have their classification put in question (Carvalho et al., 2015), and, consequently, with questionable taxonomy. The genetic divergence among morphologically indistinguishable specimens within a single “species” rises doubts on their taxonomic status, once such variations imply in cladogenesis processes (Bickford et al., 2007), which may end up in speciation. Some approaches based only on morphological data may sometimes underestimate such variation due to the phenotypic plasticity evidenced by a significant number of species. Thus, an integrative cytotaxonomic, molecular and morphological analysis is required, attempting to elucidate the real taxonomic status of polymorphic species.

Cytogenetic studies, including the molecular organization and cytogenetic mapping of repetitive DNAs might be a significant data set for the characterization of particular segments of biota, providing important information for phylogenomics (Cioffi et al., 2012). Besides, these sequences seem to escape the selective pressure that acts in the non-repetitive segments, thus representing good evolutionary markers to detect recent events of evolution, once the number and location of these sequences may reveal polymorphisms, with intra- and inter-specific variations due to rearrangements, even in conserved karyotypes (Cioffi et al., 2009; Matoso et al., 2011; Motta-Neto et al., 2012; Oliveira et al., 2015). Up to now, only two *Bunocephalus* species had cytogenetic studies already conducted, *B. doriae* and *B. coracoideus*. While the first one has 2n = 50 chromosomes, the later presents 2n = 42, in addition to a multiple X_1_X_1_X_2_X_2_/X_1_Y_1_X_2_Y_2_ sex chromosome system (Fenocchio and Swarça, 2012; Ferreira et al., 2016).

Besides, genetic data can unmask distinct populations covered by a same taxonomic status, which are identified as Significant Evolutionary Units (ESUs) by the conservation biology area. Thus, ESU corresponds to a population, or even to a group of populations, genetically distinct within a given species that contribute to biodiversity (Hey et al., 2003). The ESUs recognition is a task that requires the accordance of a group of procedures as an identification criterion.

The present study is a contribution to the biodiversity presented by *B. coracoideus* using DNA barcoding and conventional and molecular cytogenetic methodologies. It was analyzed four allopatric populations from the Amazonian hydrographic basin and the results were able to highlight a huge cryptic diversity both intra- and inter-populations, pointing out that *B. coracoideus* corresponds to a species complex.

## Materials and Methods

### Specimens

**FIGURE 1:**
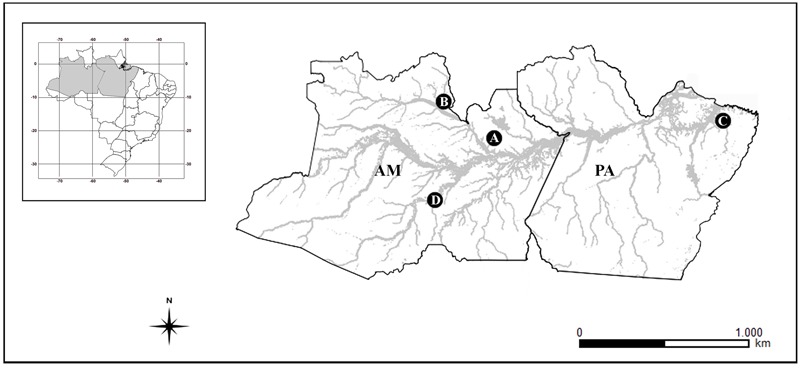
Amazonas (AM) and Pará (PA) Brazilian states map, highlighting the collection sites of the analyzed species. **(A)** ESU-A Igarapé Jundiá – Cuieiras River, **(B)** ESU-B Demini River, **(C)** ESU-C Igarapé Apeú – Guamá River, and **(D)** SEU-D_1-14_ Purus River.

Individuals of *Bunocephalus coracoideus* from four populations of distinct drainages of the Amazon River were analyzed (**Figure 1** and **Table 1**). The specimens were collected under appropriate authorization of the Brazilian environmental agency ICMBIO/SISBIO (License number 48795-1). All specimens were properly identified by morphological criteria and voucher samples were deposited in the fish collections of the National Institute for Amazon Research (INPA: *Instituto Nacional de Pesquisas da Amazônia*). The experiments followed ethical and anesthesia conducts, in accordance with the Ethics Committee for Animal Use of the National Institute of Amazon Research, under the protocol number 010/2015.

**Table 1:** Estimates of evolutionary divergence between sequences using the COI gene and the K2P model.

	ESU-A	ESU-B	ESU-C	ESU-D
ESU-A	0.2			
ESU-B	12.2	0.2		
ESU-C	10.6	4.0	0.7	
ESU-D	12.3	5.7	3.8	2.5

*Values are shown as percentages.*

### Mitotic and Meiotic Chromosomal Preparations

Mitotic chromosomes were obtained from the kidney cells according to the protocol described by Gold et al. (1990), using RPMI culture medium (Cultilab). Male testis were used for meiotic preparations, following the protocols described by Bertollo et al. (1978), with changes introduced by Gross et al. (2009). For conventional cytogenetic analysis the chromosomes were stained with 5% Giemsa solution (pH 6.8).

### Preparation of FISH Probes

The GoTaq Colorless Master Mix (Promega) was used for the polymerase chain reaction (PCR) amplification of the 18S and 5S rRNA genes and telomeric sequences, using the following primers: 18Sf (5′-CCG CTT TGG TGA CTC TTG AT-3′), 18Sr (5′-CCG AGGACC TCA CTA AAC CA-3′) (Gross et al., 2010), 5S A (5′-TAC GCC CGA TCT CGT CCG ATC-3′), and 5S B (5′-CAGGCT GGT ATG GCC GTA AGC-3′) (Martins and Galetti, 1999). The ribosomal sequence amplification cycles comprised a denaturation for 2 min at 95°C; 35 cycles of 1 min at 94°C, 30 seg. at 56°C, and 1.5 min at 72°C; a final extension of 5 min at 72°C; and a cooling period at 4°C. The primers (TTAGGG)5 and (CCCTAA)5 (Ijdo et al., 1991), were used to obtaining telomeric sequences. PCR was performed with the following profile: 4 min at 94°C; 12 cycles of 1 min at 94°C, 45 s at 52°C, and 1.5 min at 72°C; and 35 cycles of 1 min at 94°C, 1.5 min at 60°C, and 1.5 min at 72°C. The 18S rDNA and the telomeric probes were labeled with digoxigenin-11 dUTP using a DIG-Nick Translation Mix kit (Roche), while the 5S rDNA probe was labeled with biotin-14-dATP using a Biotin-Nick Translation Mix kit (Roche) according to the manufacturer’s instructions.

### Detection of Repetitive DNA Sequences by FISH

Fluorescence *in situ* hybridization (FISH) was performed according to the protocol described by Pinkel et al. (1986), with some modifications. The 18S rDNA and telomeric probes were detected with Anti-digoxigenin-rhodamin (Roche, Basel, Switzerland), while the 5S rDNA probe was detected with avidin-FITC (Sigma). Chromosomes were counterstained with DAPI (1.2 μg/ml) and slides mounted with antifade solution (Vector, Burlingame, CA, United States).

### Microscopic Analysis and Image Processing

At least 30 metaphase spreads per individual were analyzed to confirm the 2n, karyotype structure and FISH results. Images were captured using an Olympus BX50 microscope (Olympus Corporation, Ishikawa, Japan) with CoolSNAP camera and the images processed using Image Pro Plus 4.1 software (Media Cybernetics, Silver Spring, MD, United States). The chromosome classification followed the method proposed by Levan et al. (1964), with the following limits for the arms relationship (AR): AR = 1.00–1.70, metacentric (m); AR = 1.71–3.00, submetacentric (sm); AR = 3.01–7.00, subtelocentric (st); and AR > 7.00, acrocentric (a). For the number of chromosome arms [fundamental number (FN)], the metacentric, submetacentric, and subtelocentric chromosomes was considered having two chromosomal arms and the acrocentric chromosomes a single one.

### DNA Barcoding Analysis

Representatives of each population were used (**Table 1**). *Bunocephalus* cf. *aloikae, B. amaurus*, and *Amaralia hypsiura* species were used as out groups. Tissues of liver and muscle were stored in absolute ethanol for the acquisition of Cytochrome *C* Oxidase Subunit 1 (COI) sequences. Total DNA was obtained by Wizard^®^ Genomic DNA Purification Kit. The pair of primers used for the COI mitochondrial region amplification in PCR reactions was VF1_t1 (TGT AAA ACG GCC AGT CAA CCA ACC ACA AAG ACA TTG G) + VR1_t1 (CAG GAA ACA GCT ATG ACT AGA CTT CTG GGT GGC CAA AGA ATC A) (Ivanova et al., 2006). Each PCR reaction presented a final volume of 25 μl containing 1 μl of DNA template [250 ng/μl] + 1 μl of each primer [5 pM]. It was used the GoTaq Colorless Master Mix^®^ (Promega) for the PCR. The amplification cycles comprised denaturation, 2 min at 95°C; 35 cycles of 1 min at 94°C, 30 seg. at 56°C, and 1.5 min at 72°C; a final extension of 5 min at 72°C; and a cooling period at 4°C. PCR products were visualized on a 1.7% agarose gel and purified with 20% PEG (Lis, 1980). For sequencing it was used a “Big Dye Sequence Terminator v.3.1” kit (Applied Biosystems), according to the manufacturer instructions. The amplification conditions were comprised 25 cycles at 96°C for 30 seg.; 15 seg. at 50°C; and 4 min at 60°C. After the reaction, the products were precipitated and sequenced (sequencer model ABI PRISM 3100 Genetic Analyzer from Applied Biosystems/made by HITACHI).

### Sequence Alignment and Phylogenetic Analysis

Sequences with 690 pb were used to perform the barcoding analyses, by using COI gene, which were aligned using the Geneious^®^ 10.1.3 software. The distance model of Kimura 2-parameters (Kimura, 1980), was used to build a Neighbor-Joining (NJ) dendrogram and a bootstrap analyses was performed (Felsenstein, 1985) with 1,000 replicates. All the aligned sequences were translated into amino acids to detect possible alignment errors. The Maximum Likelihood (ML) model (Tamura et al., 2004) was performed to recover the phylogenetic topology. All positions containing gaps and missing data were eliminated. There were a total of 461 positions in the final dataset. Pairwise genetic distance calculations and NJ tree analysis were implemented using Molecular Evolutionary Genetics Analysis version 5 (MEGA5) software (Tamura et al., 2011) and applying 1,000 bootstrap replicates.

## Results

### Cytogenetic Data

**FIGURE 2:**
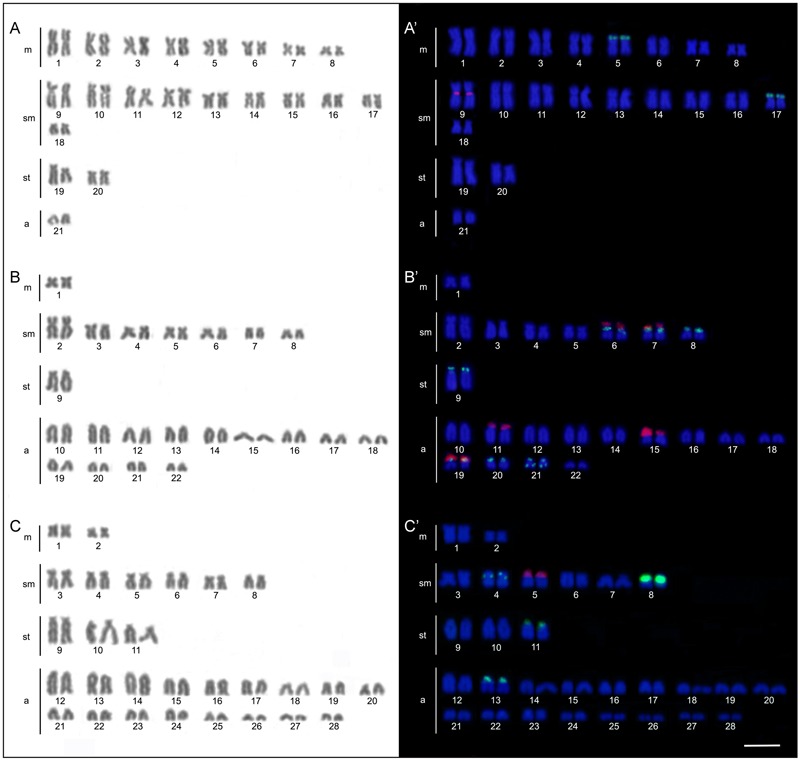
*Bunocephalus coracoideus* karyotypes. On the left, Giemsa staining; on the right. Double-FISH evidencing chromosome pairs bearing the 18S rDNA (red) and 5S rDNA (green) sequences. **(A,A′)** ESU-A Igarapé Jundiá – Cuieiras River, **(B,B′)** ESU –B Demini River (Cuieiras River) and **(C,C′)** ESU-C Igarapé Apeú – Guamá River. Bar = 10 μm.

**FIGURE 3:**
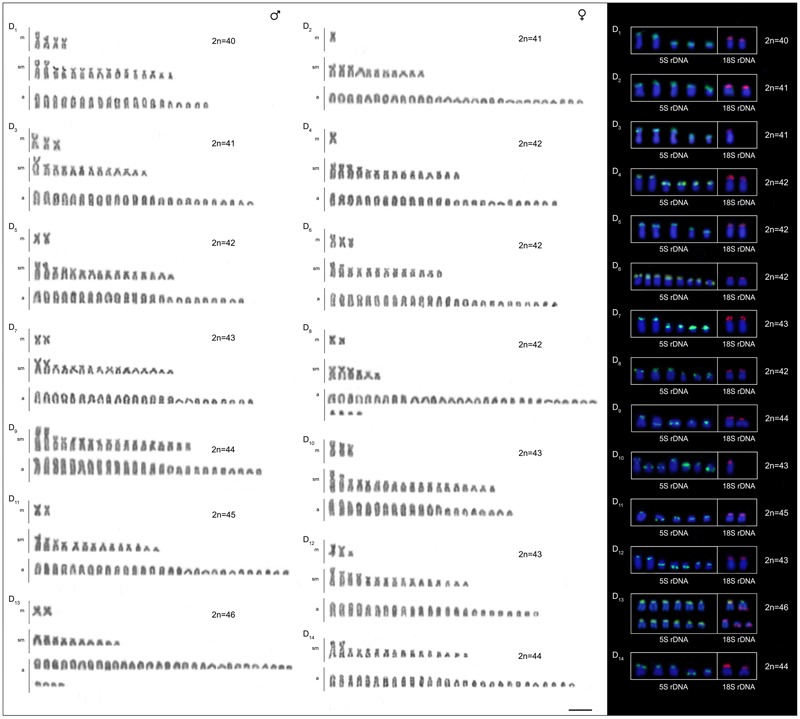
Cytotypes of *B. coracoideus* ESU-D_1-14_ Purus River, with Giemsa staining. For this ESU chromosomes were not paired, once no homeology was usually found between the correspondent pairs among the distinct cytotypes. On the right, double-FISH showing the chromosome pairs bearing the 18S rDNA (red) and 5S (green) sequences. Bar = 10 μm.

The four *B. coracoideus* populations presented distinct karyotypes, which classified as evolutionary significant units (ESUs), with the following characteristics ESU-A: 42 chromosomes (16m+20sm+4st+2a, NF = 82) from Igarapé Jundiá – Cuieiras River (**Figure 2A**), ESU-B: 44 chromosomes (2m+14sm+2st+26a, NF = 62) from Demini River (**Figure 2B**) and ESU-C: 56 chromosomes (4m+12sm+6st+34a, NF = 78) from Igarapé Apeú – Guamá River (**Figure 2C**). There was no karyotype differentiation among males and females in these populations. For the ESU-D, from Purus River, 14 distinct cytotypes bearing variant chromosomes in number and morphology were observed, with 2n varying from 40 to 46. Due to such variation, chromosomes were not grouped in pairs once homeology was not usually found among the distinct cytotypes (**Figure 3**). Significant variations were also found concerning the 18S rDNA carrier chromosomes, localization and number of sites. For ESUs A, B, and C such sequences were found in one or five chromosomes pairs, in the pericentromeric or telomeric regions, in the short or long arms (**Figure 2**). Similar results were also detected among the cytotypes of the ESU-D. However, in this case, although the telomeric position of the sites was consistent for all cytotypes, they were found in the short arms of two chromosomes in the cytotypes D_1_, D_2_, D_4_, D_5_, D_6_, D_7_, D_8_, D_9_, D_11_, D_12_ and D_14_, but in only one chromosome in the cytotypes D3 and D_10_. In turn, the cytotype D_13_ highlighted two chromosomes bearing sites in the short arms and three chromosomes in the long arms (**Figure 3**).

Likewise, the 5S rDNA also showed great variation among ESUs. Like for 18S rDNA, with distinct chromosomes carrying these sequences and with variations in number and localization on the chromosomes. ESU-A showed only two chromosome pairs carrying 5S sequences, while ESUs B, C, and D presented a higher number of these sites. Although maintaining the preferential telomeric localization, interstitial positions were also highlighted mainly among ESU-D cytotypes. In addition, the syntenic localization with the 18 rDNA was evidenced in three chromosome pairs of the ESU-B, as well as in two chromosomes of the ESU-D – cytotype D_13_ (**Figures 2, 3**).

**FIGURE 4:**
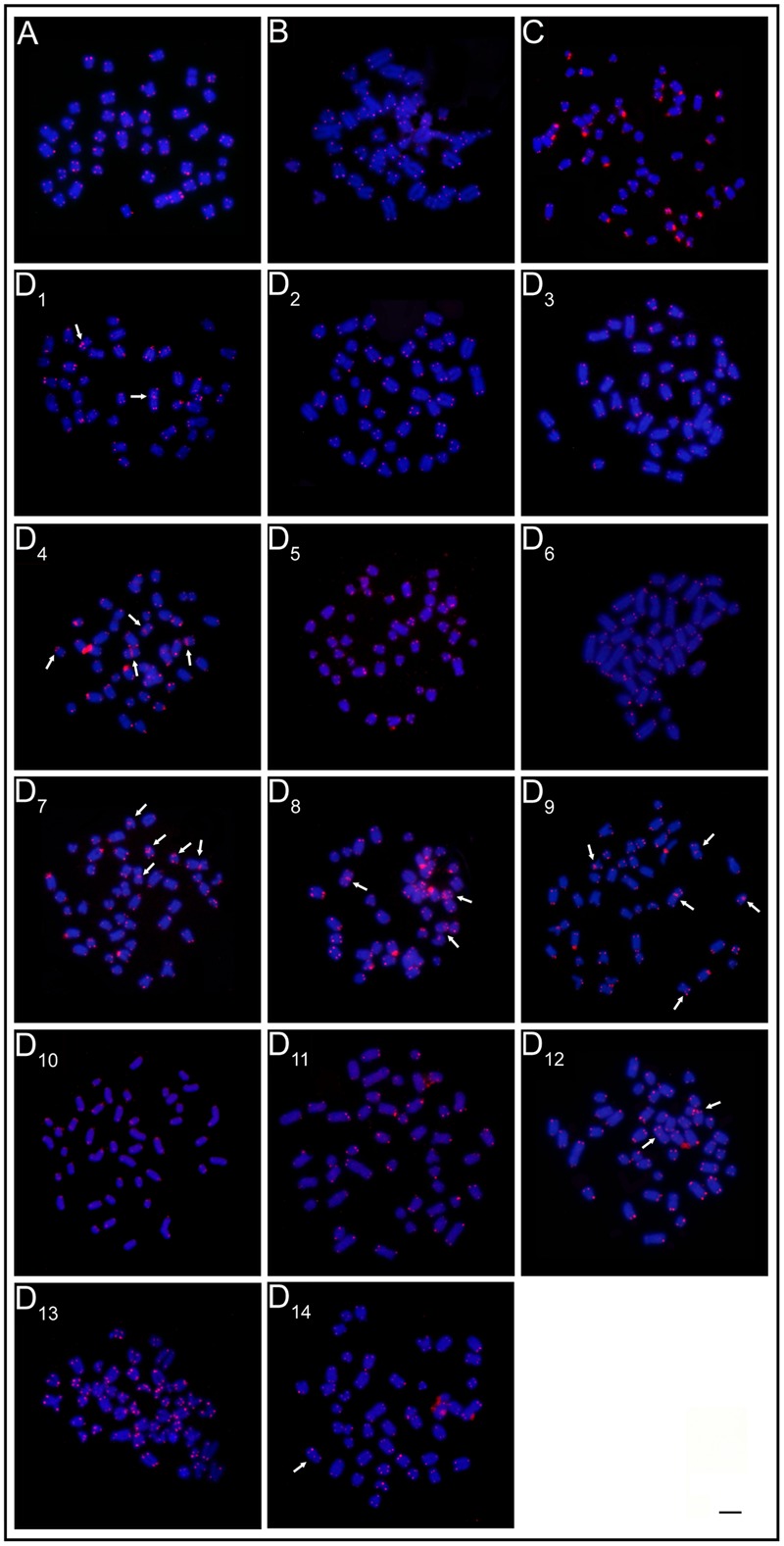
FISH with telomeric probe. **(A)** ESU-A Igarapé Jundiá – Cuieiras River, **(B)** SEU-B Demini River, **(C)** SEU-C Igarapé apeú – Guamá River, and **(D_1_-D_14_)** SEU-D_1-14_ Purus River. Arrows indicate the occurence of interstitial telomeric sites (ITS). Bar = 10 μm.

The mapping of telomeric sequences evidenced only the usual terminal marks on the chromosomes of the ESUs A, B, and C. In turn, the ESU-D exhibited additional interstitial sites (ITS) in seven cytotypes, D_1_, D_4_, D_7_, D_8_, D_9_, D_12_, and D_14_ (**Figure 4**).

**FIGURE 5:**
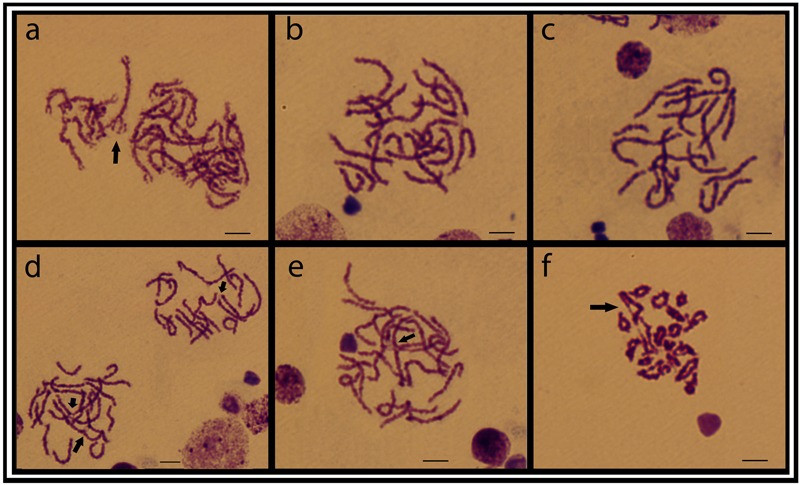
Testicular meiotic cell plates from the ESU-D in Giemsa staining. **(a)** Zigotene cells, with a possible tetravalent arrangement; **(b–e)** Aneuploid pachytene cells and arrows evidencing chiasmata formation; **(f)** Diplotene cells, with associated chromosomes likely in a chromosomal chain organization. Bar = 10 μm.

Meiotic plates from individuals of the ESU-D, showed a variable number of chromosomes corroborating the diversity found in the mitotic chromosomes. From 18 to 22 bivalents were evidenced, in addition to interstitial chiasmata and synaptic points, and probable tetravalent and chromosomal chain formations (**Figure 5**).

### DNA Barcoding Analysis

**FIGURE 6:**
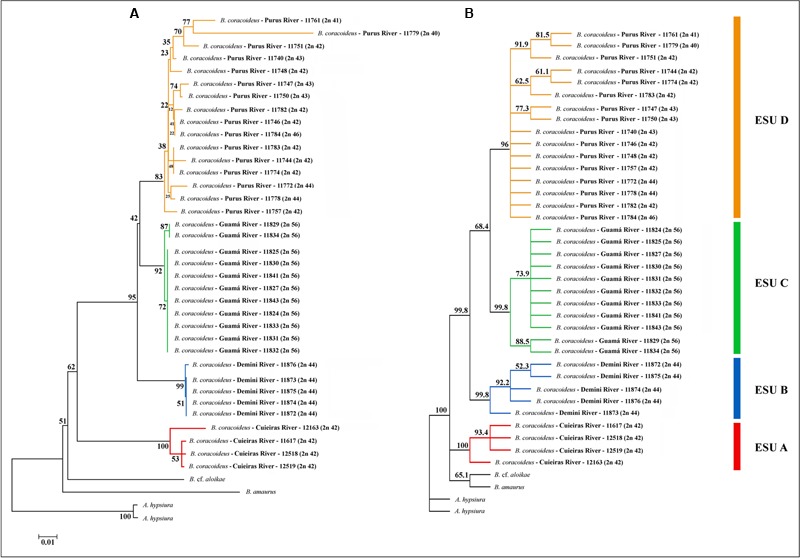
Phylogeny of *B. coracoideus* inferred by the analysis of **(A)** Maximum Likelihood (ML) and **(B)** Neighbor-Joining (NJ) using the mitochondrial gene COI. The bootstrap values for 1000 replications are evidenced above the branches.

Topologies obtained with the Neighbor-Joining (NJ) and Maximum Likelihood (ML) algorithms were congruent. The major clades were well-supported and it was confirmed that *Bunocephalus* represents a monophyletic group (**Figure 5**). Each population was also grouped as a monophyletic and well-supported clade, justifying them as four ESUs. ESU-A occupies the more basal position in relation to the other ones, and the ESUs C and D are more related to each other and with a more recent divergence (**Figure 6**).

## Discussion

**FIGURE 7:**
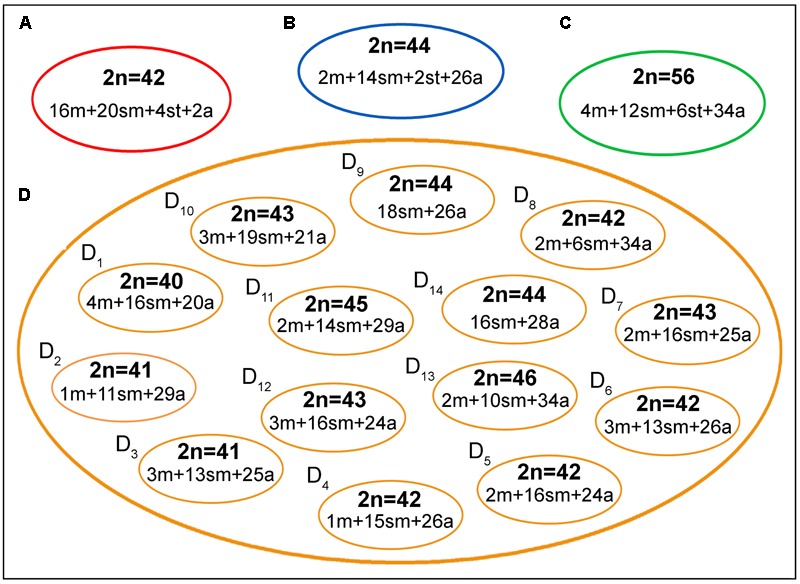
A general overview on the chromosomal heterogeneity on *B. coracoideus*. **(A)** ESU-A Igarapé Jundiá – Cuieiras River, **(B)** SEU-B Demini River, **(C)** SEU-C Igarapé Apeú – Guamá River, and **(D)** SEU-D_1-14_ Purus River.

**FIGURE 8:**
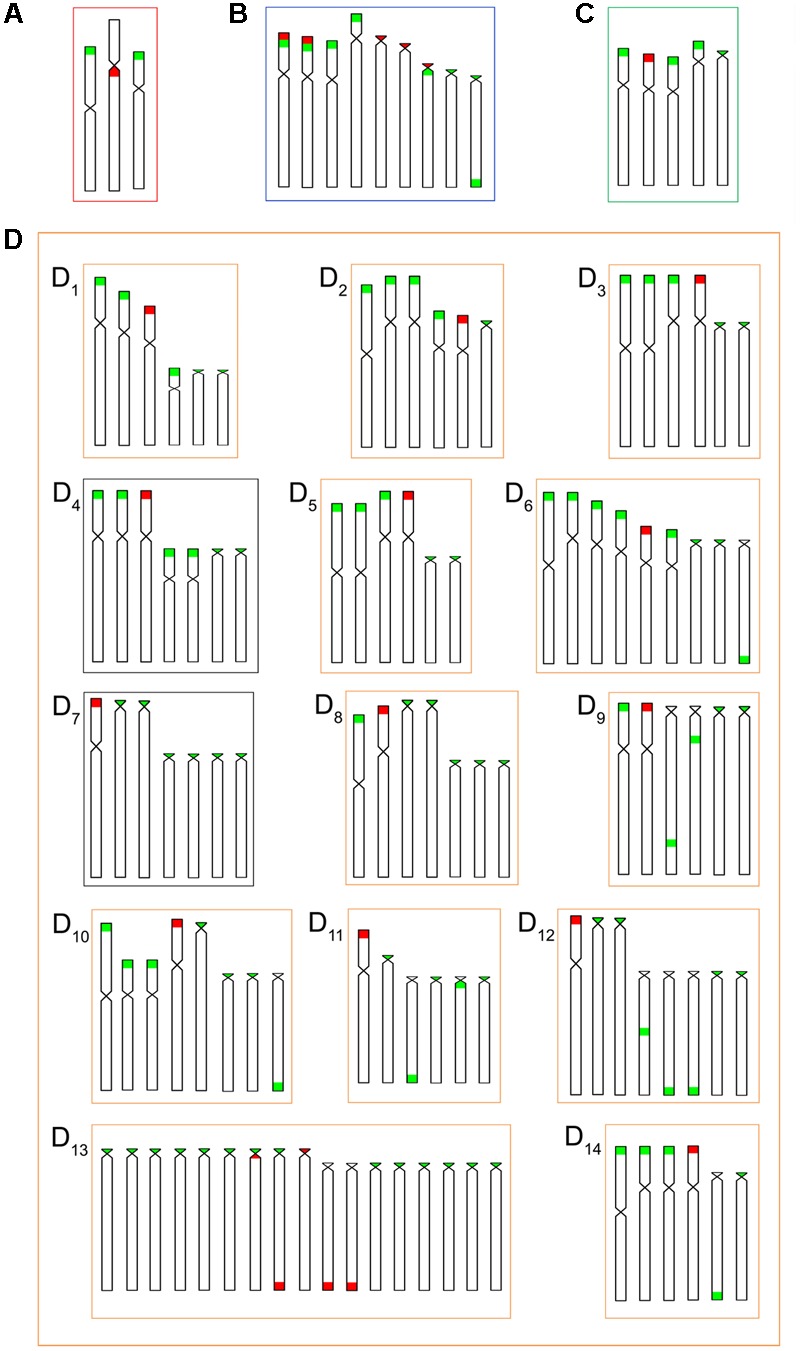
Idiograms for numbers and locations of the 18S (red) and 5S (green) rDNA sequences in the chromosomes of the *B. coracoideus* populations **(A)** ESU-A Igarapé Jundiá – Cuieiras River, **(B)** SEU-B Demini River, **(C)** SEU-C Igarapé Apeú – Guamá River, and **(D)** SEU-D_1-14_ Purus River.

The geographic distribution, genetic distances, and karyotype data indicated that each *B. coracoideus* population represents an ESU. In fact, these populations differed by conspicuous karyotypes variability, where each ESU shows specificities on their 2n, karyotype formula and ribosomal sites distribution in the genome (**Figures 7, 8**). In addition, they have possibly evolved in allopatry due to vicariant events, making their natural contact unfeasible. Oliveira and Gosztonyi (2000) proposed that the ancestral karyotype of Siluriformes contained 2n = 56 chromosomes, mainly two-armed ones. According to our phylogenetic data, ESU-A (2n = 42; 16m+20sm+4st+2a) corresponds to the firstly differentiated karyotype among the four populations analyzed. In this way, ESU- A probably retains an ancestral feature of Siluriformes by the large number of bi-armed chromosomes they have, but with the reduction of the 2n due to chromosomal fusions. In this sense, the other ESUs share a synapomorphic condition by presenting karyotypes mostly composed by acrocentric chromosomes, where pericentric inversions and/or centric fissions may have played a role. Such feature is also found in other *Bunocephalus* species, such as *B. doriae* (Fenocchio and Swarça, 2012) and *B. coracoideus* population from the Negro River (Ferreira et al., 2016), in which acrocentric chromosomes are also mainly composing the karyotype. Thus, taking in account the Oliveira and Gosztonyi (2000) proposition, it can be considered that the *Bunocephalus* ESUs A, B, and D presents a trend toward the reduction of the chromosome number in relation to other Siluriformes, while ESU-C maintained the probable ancestral 2n = 56 chromosomes.

Chromosomal rearrangements can play a role on speciation processes as they may act in the reproductive isolation (Mayr, 1995), generating an useful investigation area concerning genetic variability (Faria and Navarro, 2010), Indeed, in some distinct Neotropical fish species, such as *Hoplias malabaricus* (Bertollo et al., 2000), *Astyanax fasciatus* (Pazza et al., 2006), *Astyanax scabripinnis* (Moreira-Filho and Bertollo, 1991; Maistro et al., 2000), and *Hoplerythinus unitaeniatus* (Giuliano-Caetano et al., 2001), the chromosomal diversification raised the hypothesis that they may encompass different species under a same nomenclature.

Additionally, both number and location of rDNA sites were highly variable among ESUs (**Figures 2, 3**), highlighting their dynamic behavior in the genomes and in generation of the genetic diversity among populations. Besides, it seems that multiple 5S rDNA sites represent a synapomorphy in *B. coracoideus*, since all populations analyzed present such condition. Accordingly, ESU-A presents the lowest number of such sites (in only two pairs of chromosomes), thus representing a basal condition (**Figure 8**). Since the accumulation of repetitive sequences in particular genomic areas can cause chromosomal rearrangements (Lim and Simmons, 1994; Dimitri et al., 1997), the dynamic behavior of rRNA genes might also be linked with the huge karyotype diversity presented by this nominal species.

The occurrence of synteny between the 5S and 18S rRNA genes in the ESUs B and D (**Figure 8B**, D_13_) represents an uncommon condition among vertebrates (Martins and Galetti, 2001), once these genes are transcribed by distinct RNA polymerases, suggesting the need to be distant from each other or allocated in different chromosomes, avoiding possible harmful rearrangements between them (Amarasinghe and Carlson, 1998; Martins and Galetti, 1999). However, in Siluriformes, the syntenic condition for such both rDNA classes was already found for several species, such as *Imparfinis mirini* and *I. minutus* (Ferreira et al., 2014), *Ancistrus maximus, A. ranunculus, A. dolichopterus, Ancistrus* aff. *dolichopterus* (Favarato et al., 2016), *Hemibagrus wyckii* (Supiwong et al., 2014), *Corydoras carlae* (Rocha et al., 2016), *Panaqolus* sp. (Ayres-Alves et al., 2017) and in *B. coracoideus* (present study). Nevertheless, the evolutionary paths taking to the selection of this apparently non-advantageous condition are not revealed yet but, at first, it appears to be not a deleterious character.

An outstanding finding in our study is the huge karyotype diversity found in the ESU D. In fact, this population highlighted many varying cytotypes living in sympatry. Apparently, such polymorphism does not appear to represent effective reproductive barriers capable to impair crosses, at least among some different cytotypes, increasing the chromosome diversity inside the population. Similar intrapopulacional features were also highlighted in the Characidae, *Astyanax fasciatus*, which presented two well-defined cytotypes, 2n = 46 and 2n = 48, but with numeric and structural chromosome variants when they occur in sympatry (Pazza et al., 2006, 2007, 2008). It is noteworthy that in both cases, ESU-D and *Astyanax fasciatus*, the variant karyotypes apparently do not demonstrate deleterious phenotypic effects on the carriers. However, the hypothesis that such degree of chromosomal diversity may affect, in some way, the homeostasis of the segregation cannot be fully discarded.

To better investigate the extension of the polymorphism inside ESU-D, we extended our analyzes to the chromosomal behavior during meiosis, since it was found monosomies and trisomies in nearly all cytotypes. In order to confirm this condition, meiotic plates of three individuals were analyzed, and attested that, during meiosis I, a clear numeric variation can be observed. In fact, different bivalent numbers were found in pachytene cells of the same individual, as well as probable trivalents with synapses points. In addition, a typical tetravalent formation and an apparent chromosomal chain were also observed in zygotene and diplotene cells, respectively (**Figure 5**), and such events might have contributed for irregular segregations. It is known that chromosomal rearrangements can alter the homologs pairing during meiosis and, as a consequence, provide unbalanced gametes (Davisson and Akeson, 1993; Navarro and Ruiz, 1997; Spirito, 1998). In this way, non-disjunction events during meiosis may result in aneuploid individuals, a factor that may, at least in part, explain the polymorphic condition found in the ESU-D population. In addition, a second factor probably related to such biodiversity relies on the ecological conditions in the Purus River basin, where ESU-D occurs. This region is located in a lowland area subjected to water flooding, influenced by the seasonality of the river level (Haugaasen and Peres, 2006). These flooded forests, which appear on the rainy season, form complexes labyrinths made by tree logs, rocks and every type of vegetation common to such environments (Luize et al., 2015). This particular habitat favors fish dispersion and the consequent subpopulations segregation until their future reconnection during the dry periods.

Thus, the evolutionary scenario for the ESU-D is that chromosomal rearrangements have occurred and that geographic isolation periods, due to flood pulse cycles may have favored their fixation in the population. During the flood periods, the reestablishment of the physical connection among the previously isolated aquatic environments allowed gene flow among them and, as a consequence, the variety of the cytotypes observed among the population. This hypothesis is reinforced by the ITS found in several cytotypes, indicating the occurrence of chromosomal rearrangements (**Figure 4**).

The DNA barcoding analysis is a very informative tool for biodiversity studies. In *Salminus* fish, for example, it was evidenced eight distinct lineages increasing its current diversity, nowadays limited to four species (Machado et al., 2016). *Rhamdia voulezi* and *Rhamdia branneri*, considered synonyms of *Rhamdia quelen*, are currently argued to constitute valid species supported by karyotype, ecomorphology and morphometric data (Abucarma and Martins-Santos, 2001; Garcia et al., 2010; Mise et al., 2013; Garavello and Shibatta, 2016), as well as by the barcoding DNA analysis (Ribolli et al., 2017).

Facing the karyotype diversity found in *B. coracoideus*, the DNA barcoding methodology was also useful for analyzing the relationships among populations. In fact, this procedure is a helpful tool for analyzing the occurrence of cryptic species (Smith et al., 2008). Theoretically, the nucleotide divergences between populations of a single species (intraspecific variations) are smaller than the ones between distinct species (interspecific variations), the “barcoding gap” (Ward et al., 2005; Hajibabaei et al., 2006). Most congeneric species have showed substantial nucleotide divergences by means of this molecular marker (Hebert et al., 2003). Intraspecific divergences are rarely superior to 2%, and usually do not overcome 1% (Avise, 2000). For *B. coracoideus* the intra-population genetic distance did not overcome the value of 2%, except for the Purus population (ESU-D), which presented divergences among the sequences from 0.2 to 10.3%.

Such molecular data corroborated the karyotype diversity allowed us to infer that there is a probable ongoing sympatric speciation process within this population. From the NJ analysis, all the ESUs were supported with bootstrap values higher than 96%. The same occurred with the phylogeny based on ML, except for the ESU-D, which presented a bootstrap value of 83%, reflecting once more the karyotype variation present among the specimens of this population. However, the high value observed supports its identity as an ESU.

The ESU-A presented a mean distance of 10.6% from the other ESUs (**Table 2**), which is a value equivalent to species differences. The bootstrap value of 62% of ML between the ESU-A and the super clade including ESU-B, ESU-C, and ESU-D is much lower to the 95% ML and 99.8% NJ to grouping them. Besides, in the NJ phylogram, the ESU-A is more related to *B.* cf. *aloikae* and *B. amaurus* than to the other ESUs. In addition to its particular karyotype features, ESU-A presents a high value of allopatric speciation and the potential of being a new species. According to Avise and Walker (1999), the high divergences among the ESUs of *B*. *coracoideus* allowed us to delimitate lineages with taxonomic uncertainties in this nominal species.

**Table 2:** Aspredinidae specimens analyzed in the present study, with their respective collection places, number of individuals, diploid number (2n), and identification.

Species	Locality	Drainage	GPS data	Sampling N°	2n	ESU	GenBank access number	Voucher
*B. coracoideus*	Fazenda Dimona (Reserva PDBFF^∗^), Amazonas, Brazil	Igarapé Jundiá – Cuieiras River	2°20′59.9″ S 60°05′50.9″ W	3 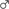 2 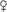	42	A	MF416164– MF416167	INPA-ICT 053204
*B. coracoideus*	Barcelos, Amazonas, Brazil	Demini River	00°23.624′ S 062°48.187′ W	2 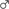 3 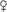	44	B	MF416168– MF416172	INPA-ICT 053205
*B. coracoideus*	Castanhal, Pará, Brazil	Igarapé Apeú – Guamá River	1°23′20.4″ S 47°59′07.5″ W	2 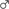 9 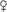	56	C	MF416173– MF416183	INPA-ICT 052188
*B. coracoideus*	Tapauá, Amazonas, Brazil	Purus River	5°37′21.7″ S 63°15′01.5′ W	2 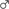	40	D	MF416184– MF416199	INPA-ICT 052185
				1 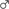 1 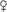	41	D		
				4 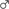 6 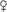	42	D		
				3 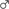 4 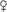	43	D		
				2 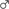 2 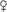	44	D		
				1 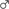	45	D		
				1 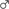	46	D		
*B. amaurus*	Castanhal, Pará, Brazil	Igarapé Apeú – Guamá River	1°23′20.4″ S 47°59′07.5″ W	1?	–	Out group	MF416200	INPA-ICT 052187
*B.* cf. *aloikae*	São Gabriel da Cachoeira Amazonas, Brazil	Curicuriari River, Igarapé Bucu – Negro River	0°14′39.0″ S 67°03′31.1″ W	1?	–	Out group	MF416162	INPA-ICT 053207
*A. hypsiura*	Cametá, Pará, Brazil	Jutuba Island – Tocantins River	02°14′46.5″ S 49°24′59.7″ W	2?	–	Out group	MF416151– MF416152	INPA-ICT 052186

*^∗^Biological Dynamics of Forest Fragments Project.*

The genetic variability and the natural selection are important conditions for evolutionary changes. Thus, understanding the neutralization of the gene flow or the locking for factors that prevent gene exchanges, such as vicariance, gene mutations and chromosomal rearrangements, are important steps to explain evolutionary processes that frequently lead to speciation (Turelli et al., 2001; Kawakami et al., 2011). Indeed, it is well-known that mutations and chromosome rearrangements can be fixated by genetic drift and, more easily, in small and isolated populations (Jesus et al., 2016), as is the case for the *B*. *coracoideus* populations here investigated. However, the great challenge for genetic biodiversity analyzes is to preserve the connection with the natural history and the species nomenclature, with consequent implications on their management and conservation (Pellens et al., 2016). In this sense, a key question that emerges is how to classify the evolutionary history of a specific population concerning their genetic diversification. In fact, the description of new species, based on genetic diversity, still finds some resistance and is not yet fully adopted. In this way, many cryptic species remain undescribed, even after their identification by genetic markers (Schlick-Steiner et al., 2007).

## Conclusion

The diversity of Neotropical freshwater fishes is still largely underestimated (Reis et al., 2016) and requires additional investigations. Nevertheless, a previous challenge remains still to be overcome: “what is a species and what new information is needed to solve this issue?” (Hey, 2001). Our study reveals a huge degree of chromosomal and genetic diversity in *B. coracoideus* and highly suggests the existence of four ESUs in allopatric and sympatric speciation processes. We believe that they were enough to reveal the occurrence of a *B. coracoideus* species complex. It indicates that new available methods, such as the genetic variability, can be definitely used in taxonomic procedures.

## Author Contributions

MF and CG performed techniques and analyzed the data; MF, JZ, and EF contributed with reagents, materials and analysis tools; MF, CG, DM, IdJ, MC, LB, JZ, and EF wrote the paper.

## Conflict of Interest Statement

The authors declare that the research was conducted in the absence of any commercial or financial relationships that could be construed as a potential conflict of interest.
